# Adsorption of Isoniazid on Aluminum Silicate Tubular Structures

**DOI:** 10.1021/acsomega.5c04344

**Published:** 2025-09-25

**Authors:** Ana Borrego-Sánchez, Carlos Gutiérrez-Ariza, C. Ignacio Sainz-Díaz

**Affiliations:** † Department of Pharmacy and Pharmaceutical Technology and Parasitology, University of Valencia, Valencia 46100, Spain; ‡ 54445 Instituto Andaluz de Ciencias de La Tierra, IACT-CSIC, Av. de Las Palmeras 4, Armilla 18100, Granada, Spain

## Abstract

Tubular morphologies were formed using chemical gardens from aluminum salts, aluminum silicate, where the external surface is formed mainly by silicate and the inner surface is mainly aluminum oxide-hydroxide. Several stages are observed during the formation of tubular aluminosilicates using the Schlieren technique. In this work, the adsorption of a bioactive organic compound on the tubular structures formed from aluminum nitrate and sodium silicate was explored, obtaining surprising results. These tubular structures exhibit a notable adsorption capacity for organic compounds and can transport pharmaceutical drugs, such as isoniazid, yielding up to 10% of the solid weight. They could also be used as an excipient in medicines. Molecular modeling studies of this adsorption process corroborated that it is energetically favorable. This material appears to be a promising candidate for the development of novel drug delivery systems for the treatment of tuberculosis.

## Graphical abstract

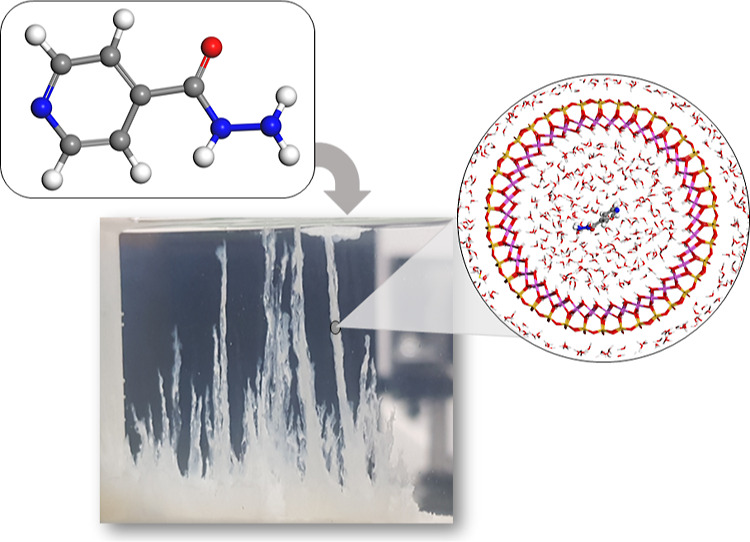

## Introduction

Chemical gardens are complex plant-like tubular structures grown in a self-organizing process under nonequilibrium conditions. When one seed of a metallic salt is immersed into an anionic dissolution, mainly a silicate or carbonate dissolution, these biomimetic structures are generated. This salt seed starts to dissolve forming a gel around it, acting as a semipermeable membrane.^1^ The water from the silicate dissolution flows in through this membrane toward the salt seed driven by osmotic pressure. Then, the solid seed continues dissolving, and the internal volume increases, rising the internal pressure. The membrane can break, forming holes, and jets of the internal fluid go up due to buoyancy forces. The contact of this fluid with the external dissolution provokes the precipitation of a solid owing to the different pH of the internal solution, forming the walls of tubes. The membrane formed around the seed is permeable to water molecules and OH^–^ anions. The concentration of OH^–^ anions in the internal fluid is lower than that in the external dissolution. These OH^–^ anions can also react with the metal cations, forming insoluble oxides-hydroxides on the internal side of the membrane and tubes. The fluid dynamics is complex within the internal chamber of these tubes, and qualitatively the behavior is similar to different inorganic cations. Previous works have used different cations as seed for the chemical garden formation.^2^ However, the differences in the solubility of these cation salts, oxides, hydroxides, or silicates can produce different ion concentrations in both dissolutions with steep chemical concentration gradients producing different behaviors with diverse morphologies, textures, and thicknesses of these tubes. These differences are based on experimental conditions, concentrations, and different solubilities of silicate and oxide-hydroxides of these cations and the permeability of the membranes. Collins et al.^3,4^ explored the formation of chemical gardens with an aluminum salt in silicate, observing a hierarchical microstructure of microtubules. Recently, we have produced aluminum silicate chemical gardens inside of an electron microscopy chamber.^5^

A natural aluminum silicate nanotube is halloysite, formed by concentric tubes with a siloxane surface oriented to the external part of the tube and an aluminol surface oriented to the internal part of the tube.^6,7^ Their structure and composition are the main factors that provide halloysites with a multitude of useful properties for their use in the health field. They are harmless, nontoxic, biocompatible, low cost, and with high adsorption properties.^8^ Accordingly, they are optimal candidates for the development of new drug delivery systems,^9^ capable of modifying the biopharmaceutical profile of drugs.^10,11^ Bioactive organic compounds can be encapsulated in the nanometric-size spaces present inside the halloysite nanotubes,^12^ and in similar tubular structures of silicates, which can be prepared by the chemical gardens formation process.^13^

Isoniazid is an antituberculosis drug used worldwide. However, this drug is used in therapy along with other drugs and for a long period of time.^14^ Hence, the development and design of new drug delivery systems for this drug is becoming an interesting issue for pharmaceutical research in order to increase its efficiency and decrease its resistances for tuberculosis treatments.^7^

One of our aims is to produce microtubes of aluminosilicate based on the chemical gardens process, exploring the mechanisms of formation of these materials using different techniques. Besides, the adsorption of the bioactive pharmaceutical drug isoniazid on the tubular structures of these materials has been investigated in this work, finding that these tubular materials can adsorb isoniazid easily. Additional atomic calculations of the adsorption of isoniazid onto the surface of a nanotube model of aluminum silicate have corroborated that this adsorption is energetically favorable. This material shows promising properties for drug delivery systems in tuberculosis treatments.

## Methodology

### Formation of Tubular Structures

Crystals of the aluminum nitrate hydrates, Al­(NO_3_)_3_·9H_2_O at analytical purity (Sigma-Aldrich, USA), were pulverized with an agate mortar and pressed into cylindrical tablets of 5 and 13 mm of diameter and 1 mm of height using a Specac Manual Hydraulic Press at 10 bar of pressure during 2 or 10 min, respectively, to avoid different shape initial conditions and to obtain a systematically uniform composition and shape. The sodium silicate dissolutions were prepared from a commercial concentrated solution composed of 27% SiO_2_ and 15% NaOH. They were diluted with Milli-Q water to several concentrations between 3 and 1 M. The tablets were placed in the silicate solutions, and the growth process was followed for at least 24 h at room temperature or as long as necessary for the complete dissolution of the salt. In some experiments, a Hele-Shaw cell was used using two transparent rectangular borosilicate plates (100 × 150 mm) separated with a silicon spacer pressing the whole system with tweezers.^15^ A metal salt tablet with a thickness the same as the gap width of the Hele-Shaw cell was first placed in the middle of the cell. The silicate dissolution was introduced slowly with a Syringe Pump LA-120 into the Hele-Shaw cell, avoiding as many perturbations as possible. The dynamics of each experiment was recorded by a Nikon D3400 digital single-lens reflex (DSLR) camera (4288 × 2848 pixels) with a Hoya circular polarizing lens filter. After that, the tubes were removed from the dissolution and dried in the air at 298 K.

For a complementary point of view on the garden growth, a dual-field-lens technique arrangement was setup.^16^ This Schlieren technique allows us to noninvasively explore the hydrodynamics acting as a guide for the chemical reaction that gives place to tubes formation. A white Mi-LED Fiber Optic LED Illuminator by Dolan-Jenner is used as the light source, and a pair of 76.6 mm Dia × 849.9 mm FL Achromatic Lenses are used to first collimate light from the source and, after it goes through the sample, focus it at the spatial filter (vertically placed knife edge) to get the characteristic light and shadow patterns related to the changes in the refraction index. A Chronos 1.4 detector from a Krontech camera was used.

### Solid Characterization Techniques

The micrographs of the samples were obtained using a Phenom Desk Scanning Electron Microscope (SEM) and an FEI Quanta 400 environmental scanning electron microscope (ESEM) at high vacuum and room temperature for the silicate experiments. Chemical analysis of solids was performed in situ in the microscope using EDX (energy-dispersive X-ray) analysis. Powder X-ray Diffraction (XRD) analyses were performed in a PANalytical X’Pert PRO diffractometer with a wavelength of 1.54 Å. Some samples were analyzed directly using a Bruker D8 DISCOVER diffractometer with a microfocus beam of variable diameter (0.1–2 mm) at a wavelength of 1.54 Å and a DECTRIS PILATUS3R 100 K-A detector. The identification of crystallographic phases in the XRD patterns was performed with the Xpowder code.^17^

### Adsorption of Isoniazid

The tubes formed with aluminum nitrate in sodium silicate 1 M, described above, were dried at room temperature. The adsorption experiments of isoniazid (Sigma-Aldrich, USA) were carried out on these solid tubes. A known amount, 15 mg, of tubes of the chemical garden was suspended into 20 mL of isoniazid aqueous solutions containing 61.73 mg (3.09 g/L = 0.023 M) of isoniazid. The suspension was stirred carefully, minimizing the breaking of tubes in an orbital shaker with a thermostatic bath for 24 h at 25.0 ± 0.1 °C. The experiment was performed three times. The resulting suspensions were filtered, and the pristine and filtered solutions were analyzed by high-performance liquid chromatography (HPLC) as described below. The difference between the pristine and equilibrium drug concentrations in both solutions corresponds to the drug adsorption in the solid tubes. Furthermore, the amount of isoniazid retained per milligram of solid was calculated. The same experiment was carried out under the same conditions using the nontubular structures of the chemical garden.

The isoniazid concentration analysis was performed using a 1260 Infinity II Agilent HPLC equipped with a quaternary pump, an autosampler, a column oven, and a UV–vis diode-array spectrophotometer. The stationary phase was a column Kromasyl C18, 5 μm, 250 × 4.6 mm (Teknokroma), and the mobile phase was a mixture of H_2_O and CH_3_CN (95:5 v/v). The flow rate was set at 0.8 mL/min with a 50 μL injection volume. A spectrophotometer detector at a 264 nm wavelength was used, and the run time for each analysis was 7 min. Data were recorded and analyzed by using software LC Open LAB HPLC 1260 (Agilent). The response of the analytical method was linear in the concentration range 5–100 mg/L isoniazid in an aqueous medium, resulting in correlation coefficients of 0.999.

Atomic models were built using the Materials Studio package applying periodic boundary conditions.^18^ The model of tubular aluminum silicate was previously optimized at quantum-mechanics level calculations based on density functional theory (DFT).^19^ The code CASTEP was used with the generalized gradient approximation (GGA) and Perdew–Burke–Ernzerhof (PBE) parameterization. The nanotube unit cell has 1292 atoms, where siloxane groups are in the external surface and the Al hydroxide groups are in the internal surface, Al_152_Si_152_O_380_(OH)_304_. The internal and external zones of the nanotube were filled with water molecules placed randomly with a density of 1 g/cm^3^ (SiAlw). The isoniazid molecule was optimized previously at the DFT level.^7^ The isoniazid molecule was placed in the center of a 3-D box creating an 3-D periodical isoniazid model (iso). Another model of isoniazid was created by placing it into the internal zone of the SiAlw model (isoSiAlw). All models were optimized by using the COMPASS force field^20^ based on empirical interatomic potentials maintaining the Al and Si atoms fixed within the Forcite code.^18^ For nonbonding, coulomb and van der Waals interactions were calculated by using the Ewald method. The adsorption energy was calculated taking into account the energies of the optimization of the models indicated in the subscripts

$$E_{ads}=E_{isoSiAlw}-E_{SiAlw}-E_{iso}$$

## Results

### Formation of Tubular Structures

In the chemical gardens growth of aluminum nitrate with silicate 3 M dissolution, one transparent tube grew forming a helical structure (Figure 1A) observing sometimes the formation of small gas bubbles and the seed osmotic balloon became also transparent (Figure 1B). Using silicate 1 M dissolution, the behavior of aluminum nitrate forms more white semiopaque tubes (Figure 1C,D). In all cases, the volume of the upper surface balloon is proportional to the initial amount of aluminum salt seed.

**Figure 1:**
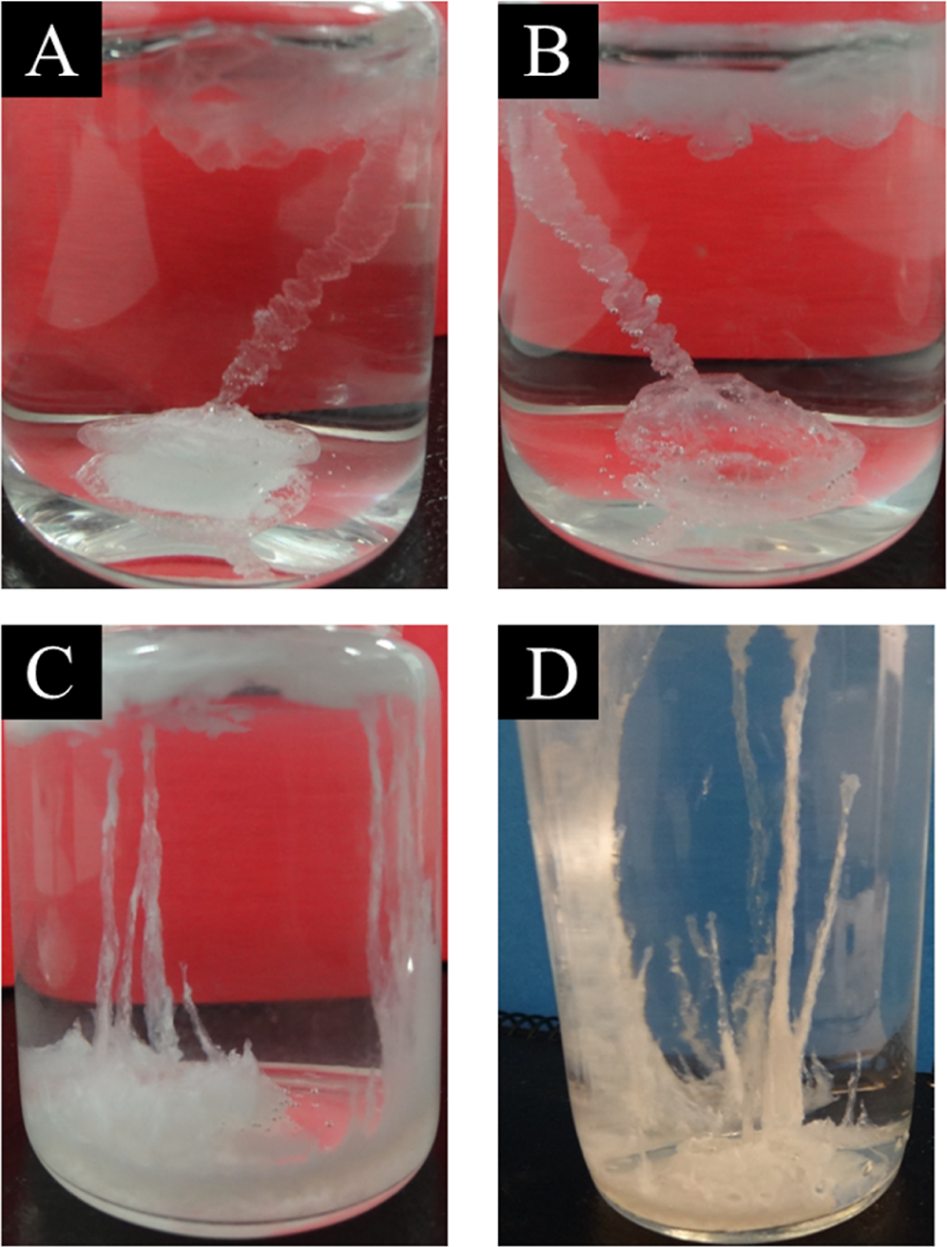
Formation of tubular structures with aluminum nitrate in sodium silicate 3 M (A,B) and 1 M (C,D) dissolutions. Pictures taken in our laboratory.

When this experiment was performed at pH 7 and 3.27 (adding HCl 0.1 M) with a 1 M sodium silicate dissolution, no reaction was observed.

For comparison and reproducibility exploration, four tablets of aluminum nitrate were placed at the bottom of a 3-D Hele-Shaw reactor of a width of 15 mm, and a sodium silicate 1 M dissolution was added slowly with a syringe pump (Figure 2). All tablets showed similar behavior, increasing the initial volume due to the swelling liquid by the osmotic pressure collapsing each other in a bottom layer. However, each tablet, within this common layer, maintains as independent membranes producing separate jets forming irregular tubes. Small differences in reaction time were observed between tablets probably due to diverse faults in the compression process of each tablet (Figure 3 and Movie M1 in the Supporting Information). In the early stages, the tubes start as transparent-translucent tubes, hinting at something invisible to the eyes occurs. The same experiment was observed by simultaneously applying the Schlieren technique. The reaction starts instantaneously when the silicate solution is added into the reactor, forming jets of a dissolution with a different refraction index than the silicate one, thus with different compositions. These jets go up by buoyancy forces, but no precipitation is detected in the initial stages. After a while, opaque solids are observed, indicating the precipitation process and the formation of the tubes (Figure 3 and Movie M2 in the Supporting Information). In some tubes, some bands with different transparency are observed. This phenomenon shows an oscillating precipitation process during the growth of the tubes.

**Figure 2:**
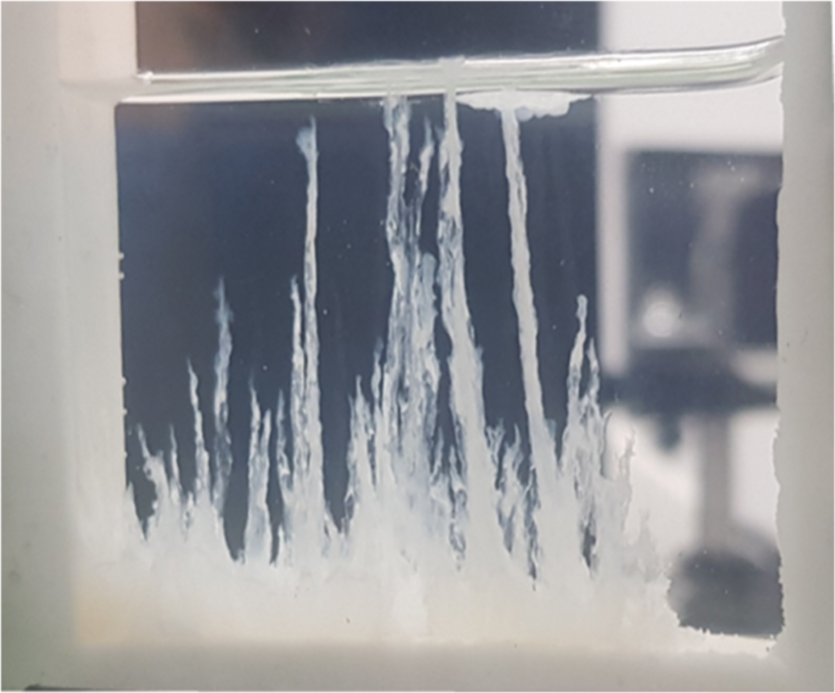
Hell-Shaw reactor used in the tubular structures formation. Picture taken in our laboratory.

**Figure 3:**
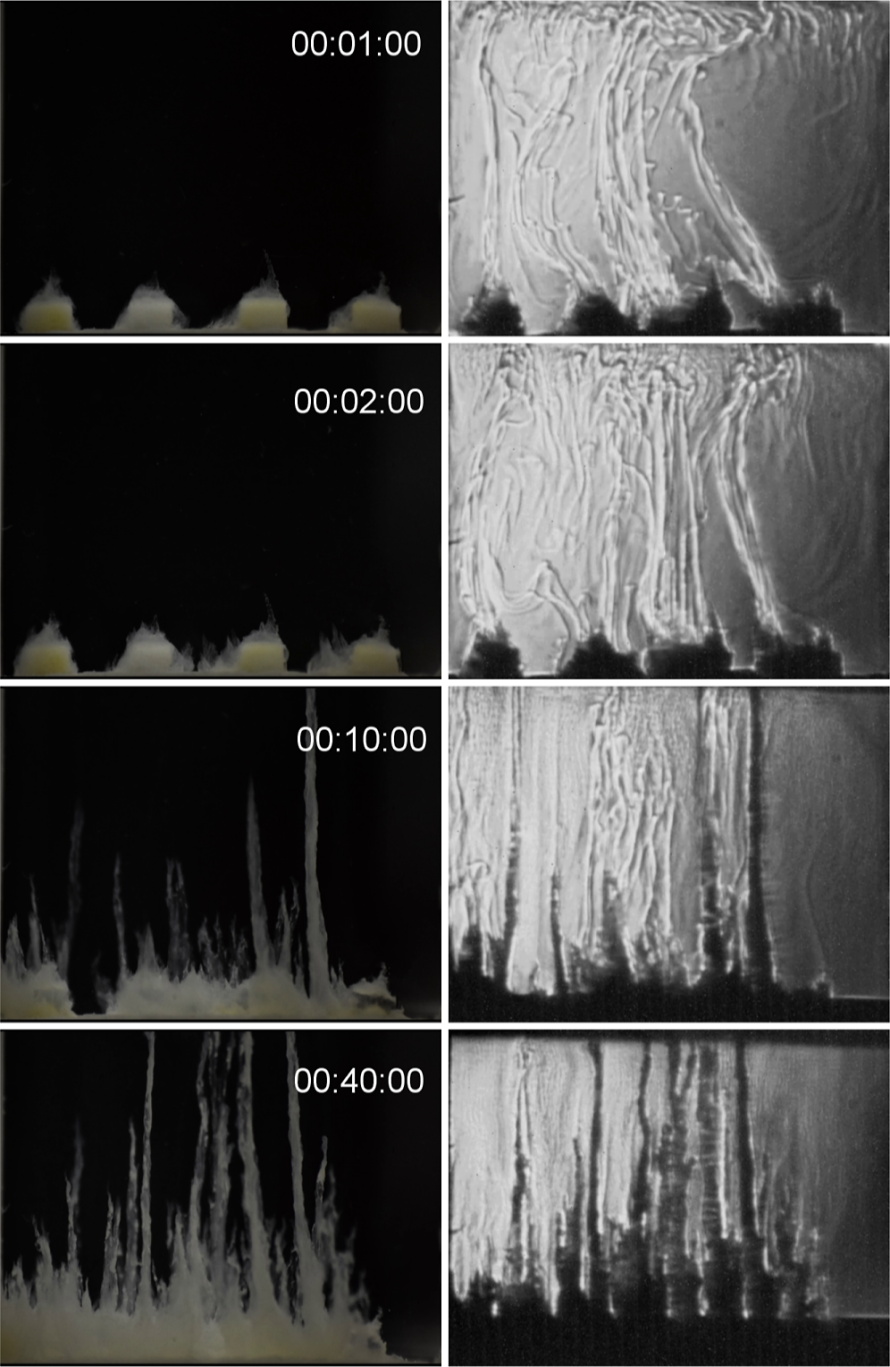
Snapshots of the chemical garden formation from aluminum nitrate tablets and sodium silicate 1 M dissolution in a 3-D Hele-Shaw reactor, optical (left column) and Schlieren (right column) pictures taken in our laboratory.

### Solid Characterization

Exploring the microstructure of these solids, we observed that the tubes have grown following one main direction with chaotic deviations. Smooth external surfaces and rugged internal surfaces are observed (Figure 4A). Small tubes are also observed with 5–10 μm of external diameter (Figure 4B,C).

**Figure 4:**
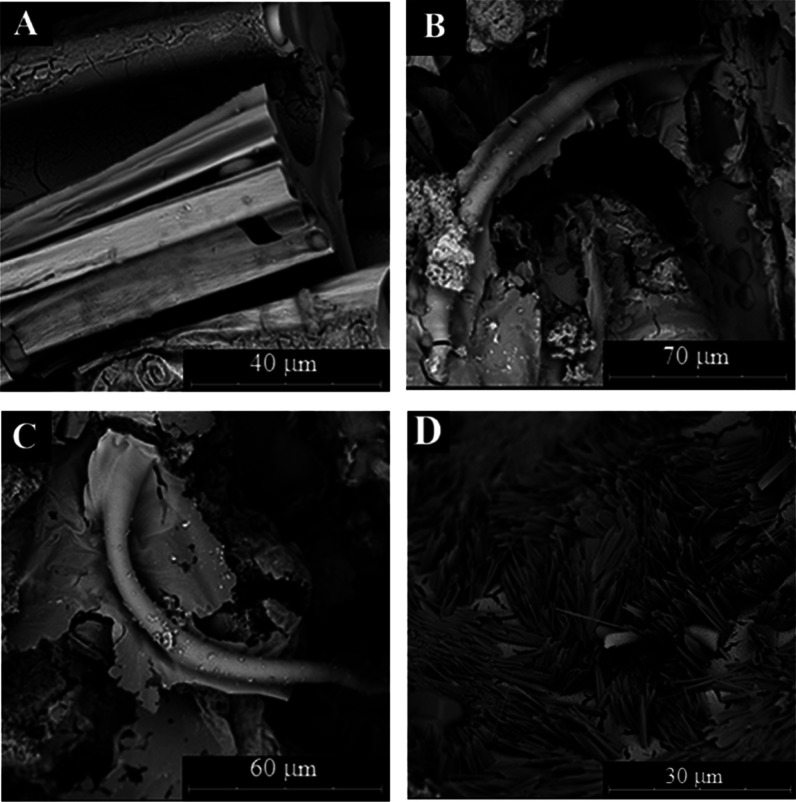
SEM micropictures (A–D) of samples obtained with aluminum nitrate salts in sodium silicate 3 M dissolution.

Several steps in the formation of tube walls can be detected in the SEM pictures of samples formed with aluminum nitrate and sodium silicate 1 M: one thin layer (1 μm) with a smooth texture, another thicker (10 μm) and rougher layer in the internal zone of the wall, and other also rough in the external surface (Figure 5A–C). Additional crystals with prismatic needles morphology are observed with different chemical compositions (different brightness) that the main wall layers. These acicular crystals are formed mainly on the internal surface, and many times, they are crossing the tube walls in perpendicular orientation (Figure 5B–D). Similar crystals with higher density were also detected using sodium silicate (3 M) (Figure 4D). These crystals can be assigned to sodium nitrate crystals.

**Figure 5:**
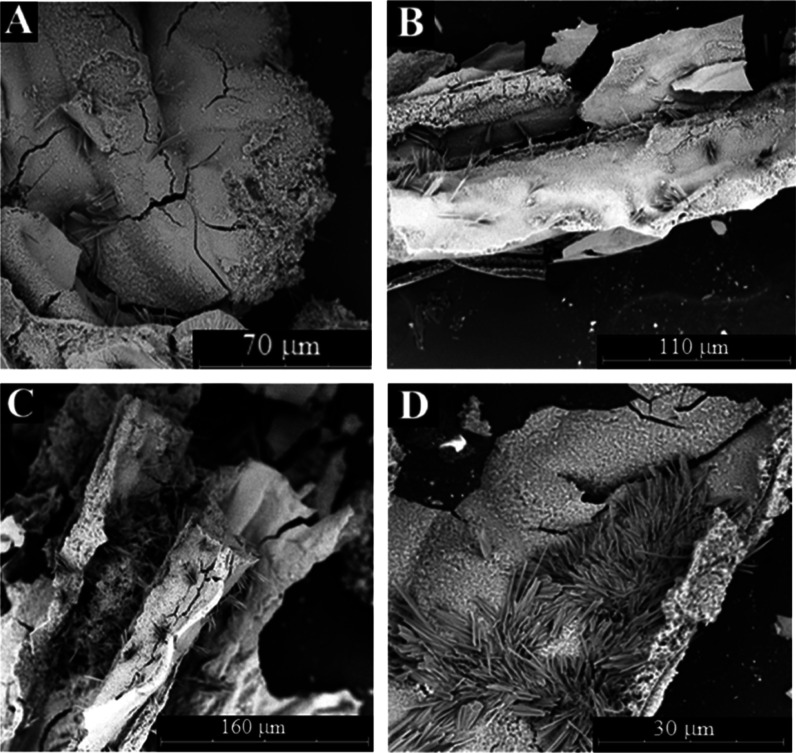
SEM pictures (A–D) of samples obtained with aluminum nitrate with sodium silicate 1 M solution.

The chemical analysis of the solids obtained from aluminum nitrate and sodium silicate showed a certain amount of N and Na along with Si and Al (Figure 6). This indicates that the Na^+^ cations have crossed the osmotic semipermeable membrane, probably due to the small size of the cation. The external surface of the tubes is mainly Si oxide with aluminum (Figure 6A). The relative amount of aluminum increased in the internal surface of tubes (Figure 6B). However, no phase frontier can be observed between Si and Al oxides and probably alumina-silicates with a gradient of aluminum content can be formed.

**Figure 6:**
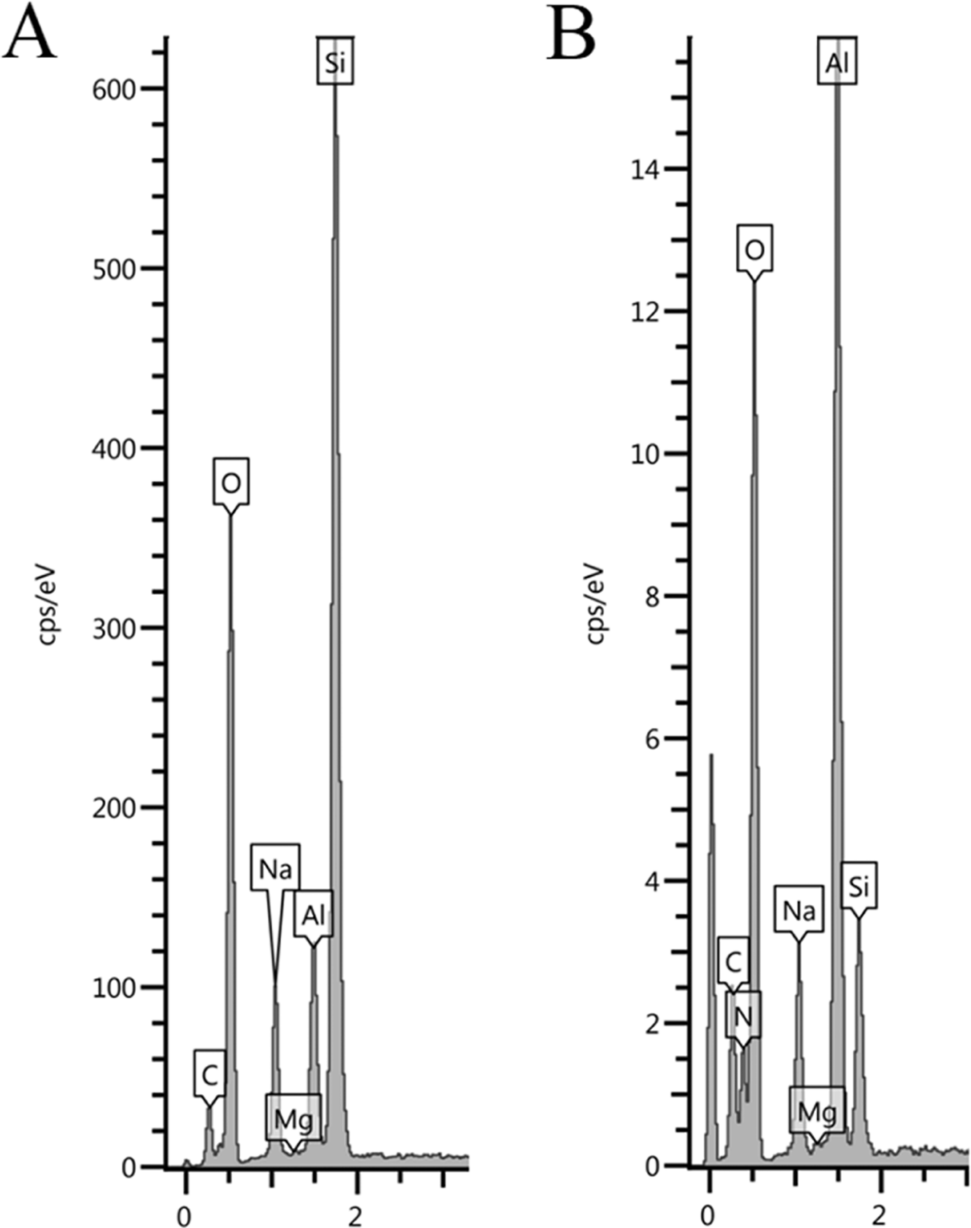
EDX chemical analysis of some solids formed from aluminum nitrate and sodium silicate, external surface (A) and internal cross-section (B).

The XRD patterns of these materials showed a highly disordered solid or a main amorphous phase in most samples. In the samples obtained with aluminum nitrate and 1 M sodium silicate, additional reflections were detected. Some points of a tube were analyzed with microfocus diffraction (Figure 7A). The rest of the tubes were milled to a powder and analyzed to get more intensity peaks. In the tube, a great amorphous phase was observed with a broad intense band (Figure 7B). Two main crystalline phases are detected as sodium nitrate with reflections at 29°, 31.8°, 38.8°, 42.3°, 47.8°, and 48.3° (2θ units) and bayerite, hydrated aluminum oxide, with reflections at 18.7°, 20.2°, 27.8°, 37°, 40.6°, and 53° (2θ units). The proportion of bayerite in the tubes is higher than in the average powder (Figure 7C).

**Figure 7:**
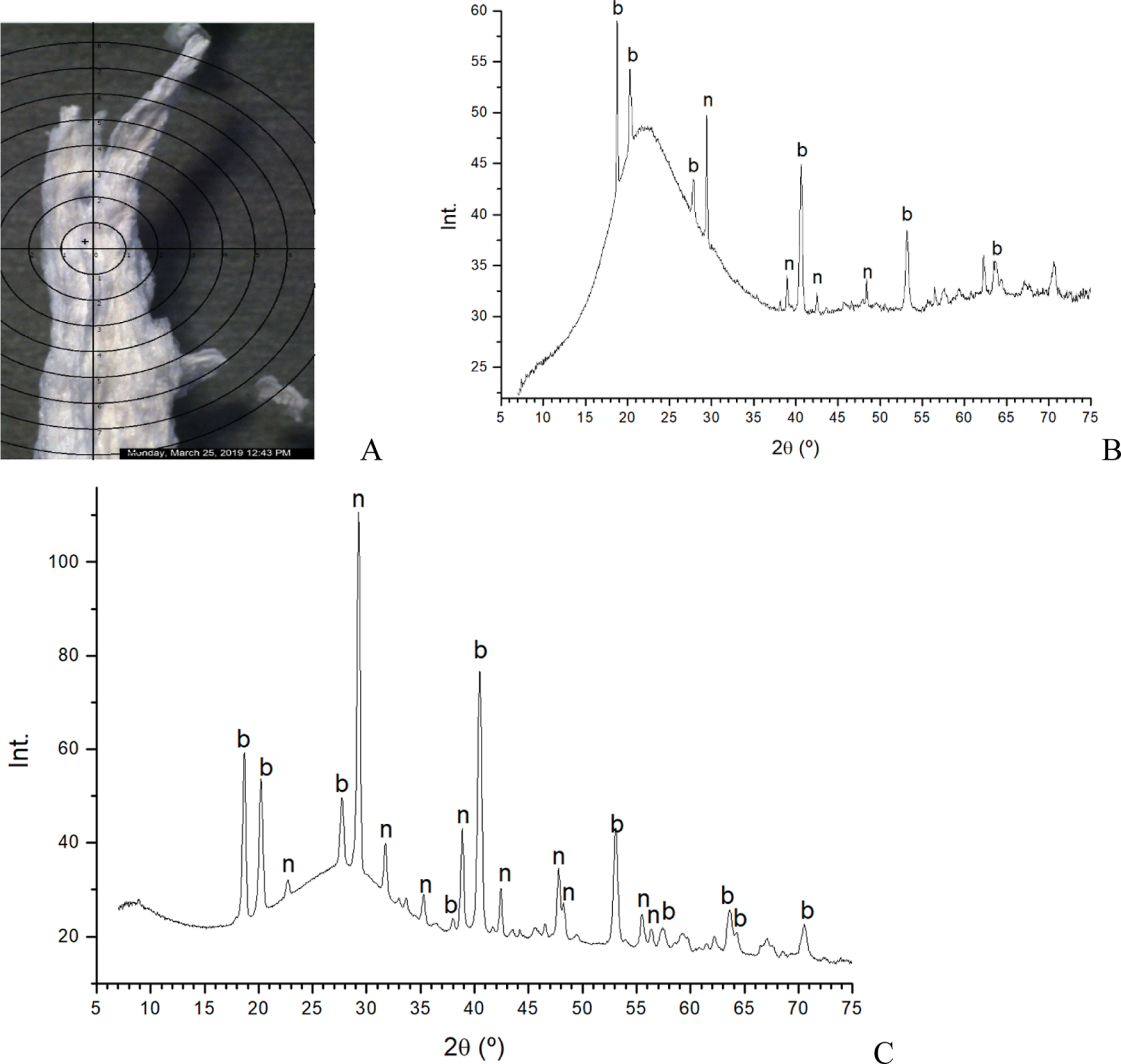
Powder X-ray diffraction patterns of chemical gardens obtained from aluminum nitrate and sodium silicate 1 M. Picture of one tube for microfocus diffraction (A), patterns of tube (B), and pattern of the whole powder (C). Reflections are assigned to b (bayerite) and n (sodium nitrate).

### Isoniazid Adsorption

The adsorption of the tuberculostatic drug isoniazid^21^ was studied on the surface of the tubular structures formed with aluminum nitrate salts and sodium silicate 1 M solution in order to explore possible future applications. The reaction time of 24 h was considered for the adsorption experiments according with previous studies of adsorption of isoniazid with other solids.^22^ After the adsorption process and with HPLC analysis, the drug adsorption onto these solids was calculated. The results showed that the total amount of isoniazid adsorbed in these tubular materials was about 2.2% w/w (average value) of that of the initial isoniazid. That means 0.1 mg of isoniazid was retained per mg of the tubular solids (a 10% isoniazid in the solid). On the contrary, no drug adsorption was observed in the other parts of the nontubular structures. This indicates that the adsorption is produced only inside the tubes and not on the external surface because this surface is partly similar to the nontubular solid that showed no adsorption.

Therefore, the isoniazid drug was effectively loaded onto the tubular structures. More comprehensive studies of adsorption isotherms and interactions of these tubular structures and drugs could provide encouraging aspects for the design of new drug delivery systems, such as isoniazid, which would improve the treatment of tuberculosis. In particular, the low cost of these materials that could act as excipients and the ease of the technique for preparing these systems are important characteristics that make these tubes promising candidates for new applications hitherto unknown in the development of drug dosage forms.

### Molecular Modeling

In the tubular structure formation, jets of internal liquid go out, and their contact with the external silicate medium produces the fast precipitation of amorphous silicon oxide (Figure 8A). After some time, the aluminum cations go up in the internal flow contacting with the Si oxide layer precipitating Al hydroxide, forming the aluminum silicate layer (Figure 8B) in a disordered way due to the fast flow and short reaction time. Our model of the aluminum silicate nanotube (SiAlw) is an ideal periodical model that can reproduce one nanoscenario where the isoniazid molecule (Figure 8C) can be adsorbed in the internal nanospace of the aluminum silicate tubes (isoSiAlw) (Figure 8D,E). The external surface of this tube is formed mainly by tetrahedra of Si oxide, and the internal surface is formed by octahedra of Al oxide-hydroxide. This is consistent with that observed in our tubular forms obtained experimentally (Figure 6). However, our models are much more ordered than the experimental one. Our model has an internal diameter of 27 Å. This tubular model is much smaller than those obtained experimentally above. Nevertheless, this model can be considered as a representation at the nanoscale of the experimental phenomenon observed above. After optimization of the isoSiAlw complex, the isoniazid molecule remains in the center of the confined nanotube. The external surface is hydrophobic, where the water molecules are pushed away. The internal surface is more hydrophilic, where the water molecules interact with the OH groups of the solid surface. The adsorption energy of isoniazid on this solid is −13.44 kcal/mol, indicating that this adsorption is energetically favorable.

**Figure 8:**
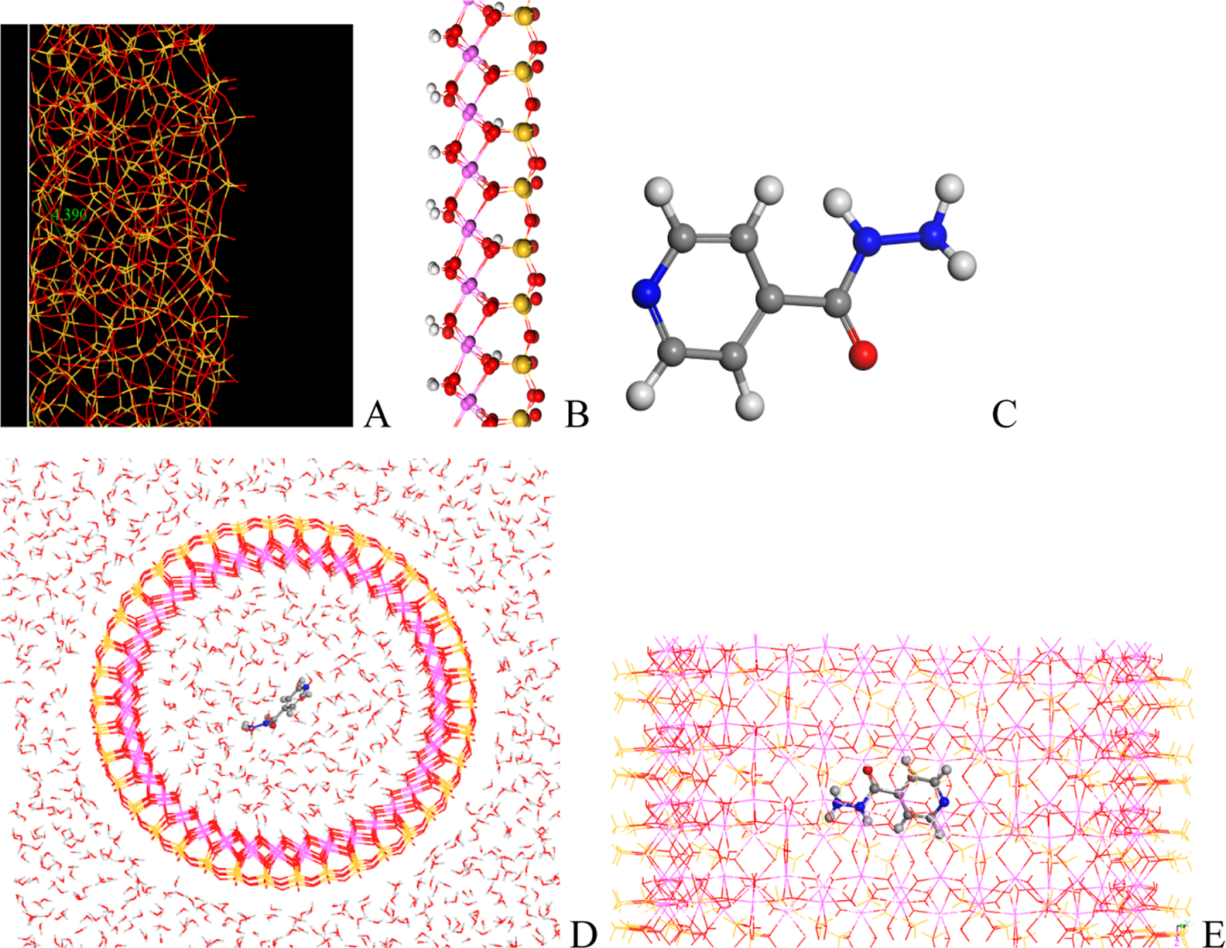
Atomic models of the tube wall of amorphous Si oxide (A), a layer of aluminum silicate (B), the isoniazid molecule (C), the nanotube in water with isoniazid adsorbed inside the tube (D), and the same from a side view (E) (water molecules were deleted for a better visualization). The H, O, N, C, Si, and Al atoms are in clear gray, red, blue, gray, yellow, and pink colors.

## Discussion

Our experiments indicate that aluminosilicate microtubes can be formed at the laboratory at room temperature following the chemical garden formation procedure. The relative behavior of each salt with the 1 and 3 M concentration of silicate is different. Nice helical wide tubes are formed in 3 M being completely different behavior than in 1 M. High pH is necessary for generating these tubular solids.

The tubular forms showed higher adsorption properties for the pharmaceutical drug, isoniazid, than the nontubular forms (mainly at the bottom part of the solids). This indicates that the porosity and infrastructure of the tubular materials are different than those in the zones close to the osmotic membrane at the bottom. Our molecular modeling calculations corroborate the adsorption capacity of these tubes for taking up isoniazid, being a process energetically favorable.

On the other hand, the morphology and chemical analysis of these tubular materials are close to that of halloysite. However, these natural minerals were formed during long geological periods of time instead of some minutes during laboratory experiments. This reaction time difference could explain the differences in size, morphology, and crystallinity observed between natural halloysite and our tubular aluminosilicates.

This adsorption of isoniazid was consistent with previous adsorption of this compound on palygorskite, a clay mineral that provides small particle size and channels for adsorption.^22^ They did not specify if the adsorption was on the external surface or into the nanochannels of 6 Å of diameter in that material.^23^ Probably, these channels are too small for the isoniazid molecule, and the adsorption will be on the external surface taking into account the high percentage of the external surface due to small particle size. Nevertheless, considering a similar concentration of isoniazid (0.023 M), the adsorption was 0.068 mg of isoniazid per mg of solid. This amount can be considered to be in the same level of our tubular solids. The same consideration can be made to the halloysite, natural nanotubes with 10–30 nm of internal diameter, and the small particle size (0.2–2 μm).^11^ Previously, this clay mineral was used for adsorption of isoniazid obtaining a similar amount of 0.1 mg of isoniazid per mg of solid for low concentrations of drug like in our work (0.023 M).^12^ Our tubular materials have greater tube internal sizes than the above clay minerals; however, the adsorption capacities are similar.

This adsorption study is the first exploration of the application of these chemical garden tubes. Our initial good results enable us to optimize several variables related with this adsorption of isoniazid, increasing the number of tubes with small internal diameter and the initial concentration of isoniazid in the future.

## Conclusions

Our work confirms that tubular structures of Al silicate can be formed from aluminum nitrate growing chemical gardens. A gradient of Al concentration is observed in these structures, being more important in the internal surfaces of the tubes. The external surface is formed mainly of amorphous silicon oxide. The Na^+^ cations of the external dissolution cross into the osmotic membranes forming nitrate salt.

These tubes provide interesting adsorption capacity for pharmaceutical drugs such as isoniazid, achieving loadings of up to 10% of the solid weight. A similar amount of isoniazid adsorption has been reported for other solids, such as palygorskite and halloysite, at low isoniazid concentrations. This work represents an initial step toward the development of modified drug delivery systems with promising features that could enhance the treatment of tuberculosis disease.

## Supplementary Material







## Data Availability

The data are available throughout the manuscript and supporting files. Additional data related with this work can be available from the corresponding author upon reasonable request to ci.sainz@csic.es

## Notes

(PDF)
Tubular structures formation from aluminum nitrate tablets and sodium silicate 1 M dissolution in a 3-D Hele-Shaw reactor (MP4)
Schlieren record of the tubular structures formation from aluminum nitrate (MP4)
