# Supplementary material for: Adsorption of Isoniazid on Aluminum Silicate Tubular Structures
Source: ACS Omega. 2025 Sep 25;10(39):45815–22. doi: 10.1021/acsomega.5c04344 (PMC12508985; doi:10.1021/acsomega.5c04344)
Supplement: Supplementary file 1 [file ao5c04344_si_001.pdf]

SUPPLEMENTARY MATERIAL

Adsorption of isoniazid drug on aluminium silicate tubular structures

Ana Borrego-Sánchez[a], Carlos Gutiérrez-Ariza[b], C. Ignacio Sainz-Díaz[b]*

[a] Department of Pharmacy and Pharmaceutical Technology and Parasitology, University of Valencia, 46100 Valencia, Spain.

[b] Instituto Andaluz de Ciencias de la Tierra, IACT-CSIC, Av. de las Palmeras 4, 18100 Armilla, Granada, Spain.

*ci.sainz@csic.es

Movie M1.- Tubular structures formation from aluminium nitrate tablets and sodium silicate 1M dissolution in a 3-D Hele-Shaw reactor.

Movie M2.- Schlieren record of the tubular structures formation from aluminium nitrate.
